# Case Report: Emergency CABG Following Failure of PTCA in a COVID-19 Patient

**DOI:** 10.3389/fcvm.2020.620610

**Published:** 2021-01-11

**Authors:** Silvia Romiti, Marco Totaro, Amalia Laderchi, Mariangela Peruzzi, Mattia Vinciguerra, Ernesto Greco

**Affiliations:** ^1^Department of Clinical, Internal Medicine, Anaesthesiology and Cardiovascular Sciences, Sapienza University of Rome, Rome, Italy; ^2^Department of Anaesthesiology, Sant'Andrea Hospital, Rome, Italy; ^3^Department of Medical-Surgical Sciences and Biotechnologies, Sapienza University of Rome, Latina, Italy; ^4^Mediterranea Cardiocentro, Naples, Italy

**Keywords:** CABG, cardiopulmonary bypass, COVID-19, SARS-CoV-2, coronary artery dissection

## Abstract

The coronavirus disease 2019 (COVID-19) pandemic outbreak, caused by severe acute respiratory syndrome coronavirus-2 (SARS-Cov-2) is affecting people worldwide representing a public health emergency. The effect of concomitant COVID-19 on patients who underwent cardiac surgery using cardiopulmonary bypass (CPB) is still undefined. Both SARS-Cov-2 infection and CPB can develop a cytokines storm and haemostatic disarrangements leading to acute respiratory distress syndrome (ARDS) and post-perfusion lung syndrome, respectively. SARS-Cov-2 infection may trigger and exacerbate post-inflammatory state after CPB resulting in higher risk of post-surgical adverse outcomes. International guidelines lack to provide standard management protocols for pre-operative COVID-19 patients requiring non-deferrable cardiac surgery intervention. We present a report of a successful coronary artery bypass grafting (CABG) emergency operation in a COVID-19 patient, who presented unstable angina and coronary artery dissection during cardiac catheterization and percutaneous transluminal coronary angioplasty (PTCA).

## Introduction

The Coronavirus disease 2019 (COVID-19) pandemic outbreak, caused by severe acute respiratory syndrome coronavirus-2 (SARS-CoV-2) represents a health emergency worldwide.

Several cases reported in literature showed, despite the most common lung impairment, a cardiovascular involvement, which reflects a complex interaction between the high capacity of Sars-CoV-2 replication and a series of manifestations, often leading to a poor prognosis. A pivotal role is played by the endothelium. Physiologically, the vascular endothelium is in charge of the maintenance of the homeostasis through a fine regulation of immune competence, inflammatory equilibrium, tight junctional barriers, hemodynamic tone, and stability as well as optimally balanced thrombotic and fibrinolytic pathways. When affected by the exacerbated inflammatory cascade triggered by Covid-19, a sequelae of events lead to an alteration of the vasomotor tone and a deregulation of clotting factors, finally exerting a key-role in the magnitude of Sars-CoV-2 infection severity.

Therefore, pre-existing conditions that affect the cardiovascular system, either acquired or congenital, may explain different expressions of systemic impairment, as seen in African American descendant populations (1). Besides, the inflammatory response triggered by Sars-CoV-2 may be responsible for the worsening of endothelial dysfunction, causing rupture of atherosclerotic plaque and ultimately acute coronary syndrome (ACS) (2). Similarly, though is still undefined, the use of cardiopulmonary bypass (CPB) in Sars-CoV-2 patients who underwent cardiac surgery may exacerbate the inflammatory response, potentially affecting outcomes.

We present a report of a successful coronary artery bypass grafting (CABG) emergency operation in a COVID-19 patient, who presented unstable angina and coronary artery dissection during cardiac catheterization and percutaneous transluminal coronary angioplasty (PTCA).

## Case Presentation

A 57 years-old female, tested positive for Sars-CoV-2 and in therapy with Azithromycin 500 mg and enoxaparin sodium 4000 UI daily, has been admitted to our hospital for chest pain.

The anamnestic collection was positive for smoking, hypertension (HTN), dyslipidaemia, and hypothyroidism.

At hospital admission laboratory tests showed high levels of LDH and high-sensitive cardiac troponin T (hs-cTnT), 257 UI/L and 0.156 μg/dL at initial assay, respectively, and 0.348 μg/dL at peak. No inflammatory markers elevations associated with COVID-19 were found (Table 1).

**Table 1:** Laboratory findings at admission to our hospital.

**Parameter**	**Values**
White blood cell count (cell/mm^3^) × 10^3^	7.85
Platelet count (cell/mm^3^)	265, 000
Lymphocytes (% white cells)	17.7
Lymphocytes count (cell/mm3) × 10^3^	1.39
Blood urea nitrogen (mg/dL)	13
Serum creatinine (mg/dL)	0.9
**Initial Troponin T hs (μg/L)**	**0.156**
**Peak Troponin T hs (μg/L)**	**0.348**
**Lactate dehydrogenase (UI/L)**	**257**
C-reactive protein	<0.1
Prothrombin time INR	1
Activated partial thromboplastin time RATIO	0.9
D-dimer (μg/L)	383
Glutamic Oxaloacetic Transaminase (UI/L)	39
glutamate-pyruvate transaminase (UI/L)	61

Non ST-elevated myocardial infarction (NSTEMI) was definitively diagnosed by electrocardiogram (EKG).

**Figure 1:**
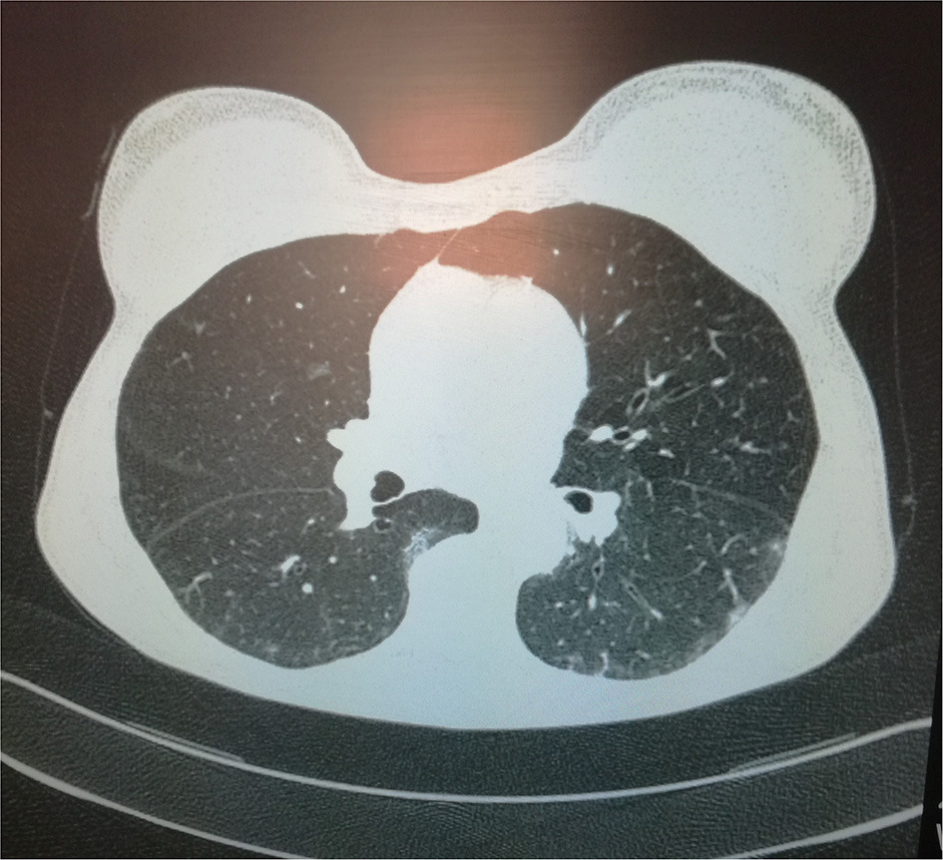
A chest computed tomographic image in the axial plane demonstrates ground-glass opacities of the lung parenchyma with bilateral and sub-pleural distribution admixed with areas of focal consolidation.

Even though the patient did not show any symptoms suggestive for COVID-19, computed tomography (CT) of the chest detected bilateral ground-glass of the lung parenchyma, in absence of pleural or pericardial effusion (Figure 1).

The patient, referred to cardiac catheterization laboratory, underwent coronary angiogram, which showed critical stenosis of both left anterior descending (LAD) coronary artery and first obtuse marginal (OM) coronary artery, and sub-occlusion of left circumflex (LCx) coronary artery.

**Figure 2:**
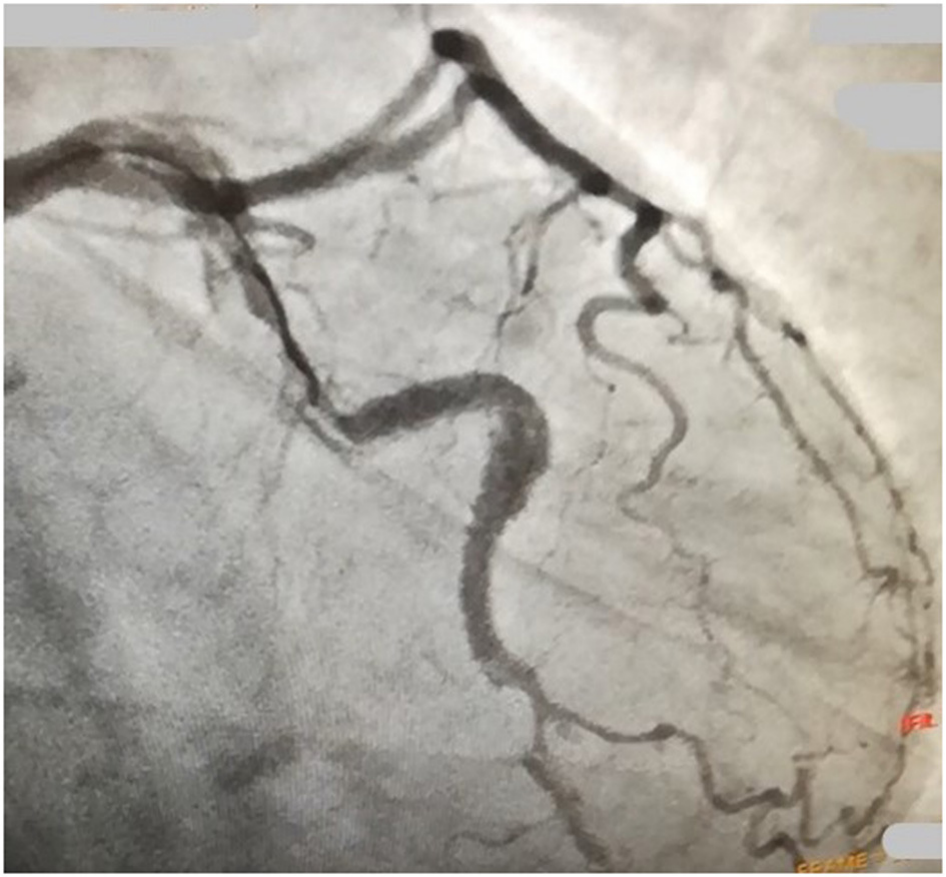
A cardiac catheterization image evidences the progression of the dissection until the circumflex artery after percutaneous coronary intervention (PCI).

During intravascular ultrasound (IVUS) study, OM coronary artery was dissected and all the efforts accomplished in order to stabilize the anatomy with Percutaneous Coronary Intervention (PCI) failed due to retrograde progression of the dissection until both LCx and LAD coronary arteries (Figure 2).

She underwent in emergency CABG surgery with cardiopulmonary bypass. She underwent routine aorta-right atrial cannulation for cardiopulmonary bypass. Myocardial protection with cold blood antegrade-retrograde cardioplegia was performed. Coronary revascularization was achieved using saphenous veins grafts [left internal mammary artery (LIMA) was not suitable because too small in size and a poor run-off] to LAD coronary artery and OM coronary artery. Both coronary vessels were extremely thin, dissected and with a very small residual lumen. Due to the hostile anatomy of the dissected arteries we disregarded the possibility of off-pump coronary revascularization. Surgery was uneventful with aortic cross-clamp and extracorporeal circulation times of 74 and 99 min, respectively.

On post-operative day 1, the patient was extubated. Transfusions of 2 units of whole blood were used to manage post-operative anemia. Post-operative chest radiographies did not show atelectasis or pleural effusion. On day 4, the patient was admitted to the ward unit. She was treated with corticosteroids (dexamethasone 10 mg for the first 10 days and then tapered over by decreasing ¼ of the dose every 48 h), empirical antimicrobials (piperacillin/tazobactam 4,5 g iv three times daily and caspofungin 50 mg iv once daily) and prophylactic anticoagulant (enoxaparin sodium 4000 UI twice daily for the first 2 weeks and then 4000 UI one daily).

Post-operative course was characterized by fever (T 38.8C°) on post-operative day 1 and 2 with a peak plasma interleukin-6 (IL-6) of 267 pg/mL (normal range 1.5–7) and by a progressive increase in D-dimer level from 665 to 4.015 μg/L.

On post-operative day 21, she was discharged after a double negative nasopharyngeal swab test for Sars-CoV-2 in excellent general condition.

## Discussion

We presented a case of ACS in a COVID-19 patient, complicated by coronary artery dissection during PTCA who underwent an emergency CABG surgery.

Although clinical signs of severe sepsis were absent, CT findings were associated with COVID-19 pneumonia.

Indeed, CT of the chest (Figure 1) showed typical features of COVID-19 pneumonia: ground glass opacities (GGOs), mixed with areas of focal consolidation with a bilateral distribution and sub-pleural predominance (3).

Interstitial lung disease increases the risks of post-operative pulmonary complications, such as acute lung injuries, acute respiratory distress syndromes (ARDS), pneumonias, atelectasis and pulmonary embolisms, among others. These significantly contribute to higher mortality rates, particularly in patients undergoing emergency surgeries (4).

Confirming these findings, an international cohort study regarding 1,128 patients across 24 countries, who underwent surgery, showed that mortality and pulmonary complications were higher in patients with perioperative Sars-CoV-2 infection. Post-operative complications (pneumonia, ARDS, request of ventilation) occurred in half of patients due to inflammatory and/or immunosuppressive responses to surgery and mechanical ventilation (5).

Therefore, despite the absence of evident symptomatology, our patient was exposed to a higher risk of post-operative pulmonary impairment.

However, pre- and post-operative prophylactic treatment of our patient might have played a crucial role in reducing the incidence of these adverse outcomes.

The patient was treated during isolation, before the onset of angina, with azithromycin 500 mg and prophylactic enoxaparin sodium 4000 UI one daily. Besides, in the post-operative period, she was treated with antimicrobial therapy, heparin, and corticosteroids.

According to the World Health Organization (WHO) guidelines (https://www.who.int/publications/i/item/WHO-2019-nCoV-Corticosteroids-2020.1) the use of systemic corticosteroids is strongly recommended for the management of severe and critical COVID-19, where the host immune response may drive the pathophysiology of the disease. Therefore, evidence suggested that the use of systemic corticosteroids reduced 28-day mortality in patients with critical and severe COVID-19 (6).

Indeed, endothelium among others is a high-specific target of Sars-CoV-2 replication, giving a paramount contribution in the dysregulation of immune system, key-mechanism associated to progressive worsening of systemic impairment caused by Sars-CoV-2 infection.

Endothelium damage, caused directly by viral replication into cells and by ACE2 downregulation, may be manifest as excessive activation of complement and abnormal cellular immune response which result in high levels of IL-1β, TNF-α, and IL-6 and haemostasis dysregulation (2, 7).

Subsequently the activation of matrix metalloproteinases and extra-cellular matrix may provide the conditions for vessel wall weakness worsening endothelial dysfunction, increasing instability of atherosclerotic plaque and vessel dissection (8). Interestingly, cases of artery dissection in young COVID-19 patients (39-, 52-, 58-years-old) without cardiovascular predisposing risk factors were recently reported (9–11), strengthening the hypothesis of COVID-19 as an endothelium disease particularly in the later stages.

In our case both coronary dissected vessels and LIMA had hostile anatomy. The LIMA was harvest but, by exploring the vessel wall, we found evidences of atherosclerosis and inadequate thickness which made it no suitable for revascularization.

Cardiac surgery itself, involving extracorporeal circulation, is frequently associated with a severe systemic inflammatory response known as systemic inflammatory response syndrome (SIRS) (12). The blood contact to non-endothelial circuit compounds promote the activation of coagulation pathways, complement factors kallikrein–kinin molecular system and a cellular immune response leading to the inflammatory storm. In addition, the response is exacerbated by synergistic role of surgical trauma, ischaemia-reperfusion injury and endotoxemia (13, 14).

SIRS may lead to an acute lung inflammation known as post-perfusion lung syndrome, in which TNF-α, IL-6, IL-8, and IL-1β have a pivotal role (15). Halter et al. (16) demonstrated a correlation between IL-6 concentrations and lung functions, showing that an increased IL-6 levels at the end of CPB correlate with impaired post-operative lung functions.

Similarly in severe COVID-19 emerging evidences suggest a correlation between increased IL-6 levels and the need for invasive mechanical ventilation (17). Furthermore, IL-6 in SARS-CoV-2 infection can be considered the key cytokine in triggering thrombotic and embolic events by increasing production of acute-phase proteins (APPs) such as C-reactive protein (CRP) and fibrinogen (2). IL-6 as a major character both involved in “cytokine storm” after CPB and severe COVID-19 pneumonia may be a potential target therapy and prognostic value in COVID-19 patient who undergo cardiac surgery at greater risk to develop post-surgical adverse outcomes.

D-dimer specially in critical COVID-19 seems to be an emerging prognostic marker of hypercoagulability (18). In our case high level of D-Dimer was treated since the beginning by Heparin and Corticosteroids.

Both CPB and Sars-CoV-2 infection trigger a “cytokine storm” which can synergistically lead to post-perfusion lung syndrome and ARDS (2, 16). In the context of pro-inflammatory state after CPB, according to the “two-hit” model, Sars-CoV-2 infection may exacerbate the systemic inflammatory response syndrome leading to acute lunge failure (19).

In order to clarify this aspect, cases of patients who underwent cardiac surgery and tested positive for Sars-CoV-2 RNA in the peri- operative period are reported.

Rescigno et al. (20) presented a case of a 63 years-old patient who underwent cardiac surgery due to severe triple vessel disease. Initially, the post-operative course was uneventful until he developed severe dyspnoea with fever leading to hypoxia. The bronchial-alveolar lavage was checked, resulting positive for Sars-CoV-2 RNA. He died on post-operative day 9 due to severe COVID-19 pneumonia.

Similarly, Fukuhara et al. (11) reported a case of acute type A aortic dissection complicated by acute ARDS, in a patient initially not suspected for, but post-operatively diagnosed with COVID-19. Despite uneventful intraoperative course, the patient, not affected by pre-existing comorbidities, developed rapidly progressive respiratory failure and expired due to multiorgan failure.

On the basis of these reports, we can hypothesize that undiagnosed COVID-19 infection might have precipitated inflammatory response after cardiac surgery.

Yandrapalli et al. (21) reported for the first time a successful coronary artery bypass operation in a pre-operatively confirmed SARS-CoV-2 infection. The authors performed pre-operatively laboratory testing for common inflammatory markers and CT findings associated with COVID-19 to identify active Sars-CoV-2 infection. Since both of them resulted negative they proceeded with the intervention. The peri- and post-surgical course was uneventful and on post-operative day 6 the patient was discharged at home.

These cases underline the importance of diagnostic testing and screening for Sars-CoV-2 in patient who will undergo cardiac surgery especially for active COVID-19 infection. It is also mandatory the need to have strict management protocols for these patients who undergo cardiac surgery in cardiopulmonary bypass due to the elevated risk of “cytokine storm” and post-operative pulmonary impairment. Systemic corticosteroids may have a potential pivotal role in the management of these patients; as reported by Xiang et al. (22) they improve severe COVID-19 by reducing IL-6 levels and activating ACE2. Interesting findings from a randomized control trial evidenced the effectiveness of corticosteroids during CPB to reduced IL-6 expression (*p* < 0.001) until 48 h after surgery compared to controls and extracorporeal hemadsorption (23). Others anti-inflammatory strategies, in severe COVID-19 undergoing cardiac surgery, could be the use of leukodepleting filters or IABP-induced pulsatile perfusion (24, 25) as in the past the use of technical alterations such as heparin coating of the CPB circuit (26).

We reported the first successful On-Pump CABG in emergency in COVID-19 patient without post-operative pulmonary complications despite pre-operative CT findings of COVID-19 pneumonia.

The specificity of our case lies in the emergency context in which the intervention was performed, highlighting the importance of providing an adequate management protocol especially for COVID-19 patients requiring acute non-deferrable surgical care and also the obligation to be more cautious during PTCA in these high risk patients.

The pre- and post-operative treatment with heparin and corticosteroids might have been crucial to reduce the incidence of pulmonary impairment, mortality rate, and other adverse outcome frequently observe in COVID-19 patients who underwent surgery as reported in several studies.

Doglietto et al. (27) in a cohort study conducted from February 23 to April 1 2020 in the Spedali Civili Hospital (Brescia, Italy) reported a higher pulmonary (OR, 35.62; 95%CI, 9.34–205.55), thrombotic complications (OR, 13.2; 95%CI, 1.48-∞) and 30-days mortality rate [odds ratio (OR), 9.5; 95%CI, 1.77–96.53] after surgery in COVID-19 patients compared with control.

The effectiveness of broad-spectrum empirical antimicrobials in patients with severe COVID-19 is still unclear, due to the low prevalence observed of acute co-infections or secondary infections coinciding with COVID-19 (28). The WHO guidelines regarding prevention of complications in hospitalized and critically ill patients with COVID-19 recommend empiric antimicrobial only with clinical suspicion of a bacterial/fungal infection for the shortest time possible to avoid an increase in antibiotic resistance (WHO/2019-nCoV/clinical/2020.5). In our case due to the onset of fever on post-operative day 1, empirical antimicrobials therapy with piperacillin/tazobactam 4,5 g iv three times daily and caspofungin 50 mg iv once daily was started with blood cultures obtained first.

No clinical data in literature are available to evaluate the real risk in the subgroup of COVID-19 patient underwent cardiac surgery and CPB to develop post-operative acute co-infections or secondary infections. International guidelines lack to provide standard protocols for pre-operative COVID-19 patients requiring acute non-deferrable cardiac surgery interventions.

## Conclusion

We need strictly management protocols to face this new healthcare environment especially for critically ill COVID-19 patients at greater risk of post-surgical morbidity and mortality: in the context of pro-inflammatory state after CPB, Sars-CoV-2 infection may trigger and exacerbate the systemic inflammatory response syndrome leading to acute lung failure increasing the risk of “citokine storm” and post-operative pulmonary impairment. Further studies are needed to evaluate this risk and the potential of corticosteroids treatment in the perioperative period, specially for patient with findings of COVID-19 pneumonia or active SARS-CoV-2 infection requiring non-deferrable cardiac surgery intervention.

## Data Availability Statement

The original contributions presented in the study are included in the article/supplementary materials, further inquiries can be directed to the corresponding author/s.

## Ethics Statement

Ethical review and approval was not required for the study on human participants in accordance with the local legislation and institutional requirements. The patients/participants provided their written informed consent to participate in this study. Written informed consent was obtained from the individual(s) for the publication of any potentially identifiable images or data included in this article.

## Author Contributions

Each author has contributed significantly to this work. EG performed the conception and design of the study and final approval. SR performed the acquisition and interpretation of data and wrote manuscript. MV performed the interpretation of data and wrote the manuscript. MT, AL, and MP revised it critically for important intellectual content. All authors contributed to the article and approved the submitted version.

## Conflict of Interest

The authors declare that the research was conducted in the absence of any commercial or financial relationships that could be construed as a potential conflict of interest.
